# Experimental and Theoretical Studies of the Thermal Contact Conductance for Bundles of Round Steel Bars

**DOI:** 10.3390/ma16216925

**Published:** 2023-10-28

**Authors:** Rafał Wyczółkowski, Vazgen Bagdasaryan, Dominika Strycharska

**Affiliations:** 1 Department of Production Management, Czestochowa University of Technology, Armii Krajowej 19, 42-200 Czestochowa, Poland; dominika.strycharska@pcz.pl; 2 Institute of Civil Engineering, Warsaw University of Life Sciences—SGGW, Nowoursynowska 166, 02-787 Warsaw, Poland; vazgen_bagdasaryan@sggw.edu.pl

**Keywords:** heat treatment, steel bars, bar bundle, effective thermal conductivity, thermal contact conduction, thermal contact conductance, guarded hot-plate apparatus

## Abstract

The paper presents investigations devoted to the analysis of the thermal contact conduction in a bundle of round steel bars. The phenomenon can be expressed quantitatively with the use of thermal contact conductance (*h_ct_*). The starting points for the presented analysis were the results of the experimental measurements of the effective thermal conductivity. The measurements were performed for samples of a medium in the form of flat packed beds of bars with three different arrangements: staggered, in-line, and crossed and four bar diameters: 10, 20, 30, and 40 mm. Next, a mathematical model was developed, thanks to which the values of the *h_ct_* coefficient were calculated for the analyzed cases. This approach consists in analyzing thermal resistances in the medium model, which is defined with an elementary cell. It was established that the value of the *h_ct_* coefficient in the temperature range of 50–600 °C changes within the range of 50–175 W/(m^2^·K), and it decreases with an increase in the bar diameter. The final effect of the present study was to develop generalized approximation equations describing changes in thermal contact conductance in the heated bar bundle simultaneously in the temperature and bar diameter function.

## 1. Introduction

**Figure 1:**
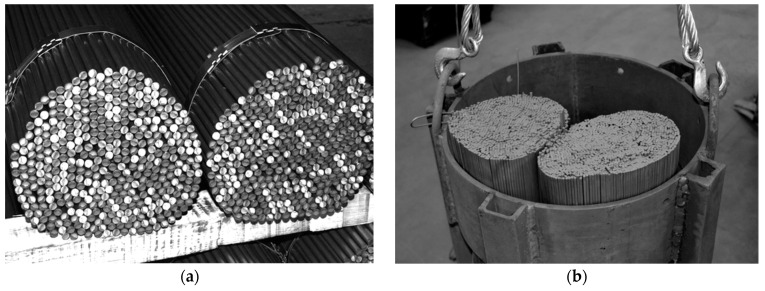
Bar bundles as an example of a steel porous charge: (**a**) a general view of a bundle showing its granular structure and (**b**) bundles prepared for heating in a soaking furnace.

Heat transfer phenomena in low-porosity granular media have been the focus of many scientific and engineering investigations [1,2,3,4,5,6,7]. Bundles of steel bars encountered in metallurgical heat treatment processes are a specific example of such media [8,9]. The bundles of bars presented in Figure 1 are a kind of steel porous charge [10,11].

From the macroscopic point of view, the geometry of a bundle can be described with two dimensions: length (*l_bn_*) and diameter (*d_bn_*). The *l_bn_* dimension is usually bigger than the diameter *d_bn_* by one order of magnitude. This disproportion means that the speed of temperature changes during the bundle heating and cooling are governed by the thermal processes, which take place in radial direction. In this plane, this charge is characterized by discontinuity of the solid skeleton. For this reason, the basic thermal property of a bundle is the effective thermal conductivity (*k_ef_*). Knowledge of the value of the *k_ef_* coefficient is indispensable to efficiently control such heat treatment of a charge. Model calculations are one method of determining the value of *k_ef_*. In the literature on heat transfer in porous media, there are a number of models that are characterized by different levels of complexity [12,13,14,15,16,17,18]. The simplest models only take into account heat transfer in individual phases (solid and gas ones), which are expressed with the use of the so-called primary parameters. These parameters include the thermal conductivity of a solid skeleton (*k_s_*), gas (*k_g_*), and medium porosity (*φ*) [19,20]. More complex models also comprise additional parameters (called secondary parameters), which make it possible to take into account the influence of factors such as the mean size of grains or voids, thermal radiation, and thermal contact conduction between adjacent grains [21,22]. As has been shown based on the authors’ own investigations, only the models which take into account the phenomenon of thermal contact conduction can be applied to calculate the effective thermal conductivity of bar bundles [23]. But in the calculation of the effective thermal conductivity of steel bars with the use of such models, the correct description of the thermal contact conduction phenomena is a very difficult problem. Therefore, knowledge of thermal contact conduction in the bar bundle is critical to model its effective thermal conductivity. This mechanism of heat transfer is quantified by the thermal contact conductance *h_ct_*, the unit of which is W/(m^2^·K). The issue of thermal contact conduction is still a current and actively researched topic. Several significant results in this field from recent years can be found in the works of [24,25,26,27]. This paper describes experimental investigations concerned with determining the *h_ct_* coefficient for bundles of round bars made of low-carbon steel.

## 2. Materials and Methods

**Figure 2:**
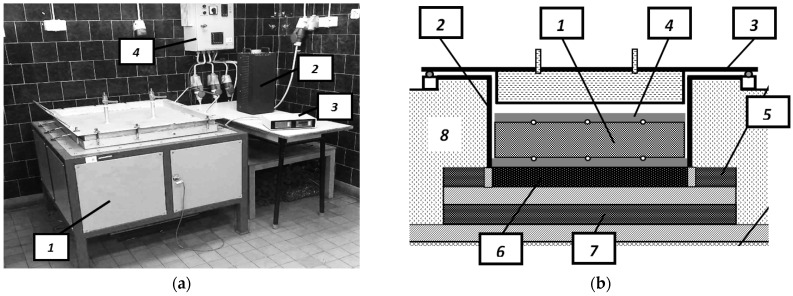
Testing stand for measuring the effective thermal conductivity: (**a**) general view of the stand: (1) heating chamber, (2) autotransformer, (3) data logger with temperature meter, and (4) control unit of main and guarded heaters; (**b**) scheme of the heating chamber: (1) investigated sample, (2) retort with a hot plate, (3) heating chamber cover with a cooler, (4) cold plate, (5) side guarded heater, (6) primary heater, (7) bottom guarded heater, and (8) thermal insulation.

In order to determine the value of the *h_ct_* coefficient, an original research methodology was used, which is based on the analysis of stationary heat transfer in flat, packed beds of steel bars. The starting points for the analyzed problem were the measurements of the effective thermal conductivity performed on a custom laboratory stand. This stand operates on the principle of a guarded hot-plate apparatus in a one-sided mode described in the ASTM standards [28,29]. Its general view is shown in Figure 2a. The main component of this stand is the heating chamber, which is schematically shown in Figure 2b.

The measurement method consists of applying a stationary, one-dimensional heat flux (*q*) between the lower and upper surfaces of the specimen. Once a stable condition is achieved, the temperatures at the lower surface (*t_lo_*) and the upper surface (*t_up_*) are recorded. In a manner akin to the characterization of thermal conductivity for solid materials (*k_s_*), the effective thermal conductivity is established as [30,31]:

(1) $$k_{ef}=\frac{q\;l_{sp}}{t_{lo}-t_{up}},$$

where (*l_sp_*) is the dimension of the sample in the direction of heat flow, denoted as is influenced by factors such as the diameter of the bar, the number of layers in the sample, and their arrangement.

The specimens under examination were positioned at the bottom of a rectangular steel retort with internal dimensions at the base measuring 400 mm × 400 mm. A primary heater with the same transverse dimensions of 400 mm × 400 mm was situated beneath the retort. The heat emitted by the primary heater was entirely transmitted to the sample, ensured by the operation of side- and bottom-guarded heaters. The power of the primary heater could be manually adjusted using an autotransformer, providing control over the measurement temperature. Additionally, a specialized control system automatically regulated the power of the guarded heaters.

Temperature measurements on the lower and upper surfaces were carried out at five opposing locations, using 0.5 mm K-type sheathed thermocouples TP-201 [32], which were connected to a multichannel data logger WRT-9 Box [33]. The thermocouples responsible for recording the bottom surface temperature of the sample were attached to the retort’s bottom, which functioned as the hot plate. In addition, the thermocouples responsible for measuring the top surface temperatures were affixed to a steel plate. This element had a thickness of 15 mm and dimensions of 390 mm × 390 mm, covering the sample and serving as the cold plate.

The heat flux (*q*) within the sample was calculated as the ratio of the heat flux rate (*Q*) emitted by the primary heater to its surface area (*A_ht_*). The value of *Q* was considered equal to the current power (*P_cu_*) supplied to this heater. *P_cu_* was measured using a 3-phase power network meter N14 [34]. Measurements were conducted for each sample with different power settings for *P_cu_*, ranging from 200 to 3200 W. This range of power settings allowed for the determination of temperature variations in the coefficient *k_ef_*. For a more comprehensive understanding of the testing setup and measurement procedure, additional information can be found in the paper written by [35].

**Figure 3:**
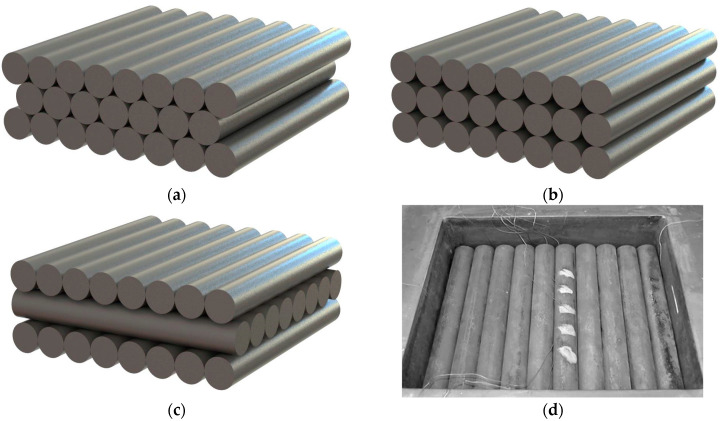
Investigated samples: (**a**) a sample with a staggered arrangement, (**b**) a sample with an in-line arrangement, (**c**) a sample with a crossed arrangement, and (**d**) one of the samples made of 40 mm bars inside the heating chamber.

The measurements were performed for 12 samples with four different bar diameters (10, 20, 30, and 40 mm) and three arrangements (staggered ST, in-line IL, and crossed CR) shown in Figure 3. The bars used for the tests were made of S235JRH steel grade, and their chemical composition and mechanical properties are described in the EN 10210-1-2006 standard [36]. According to this document, the maximum element contents in this steel were as follows: C 0.2%; Mn 1.4%; P 0.04%; S 0.04%; and N 0.009%.

For each sample, experiments were made for the temperature range of 50–600 °C. As can be seen, samples with individual types of arrangement differ in geometrical conditions of contact between adjacent bars. This differentiation of course affects the intensity of thermal contact conduction phenomena. Therefore, comparison of the results obtained for various sample types provides information on the problem analyzed in the study.

**Figure 4:**
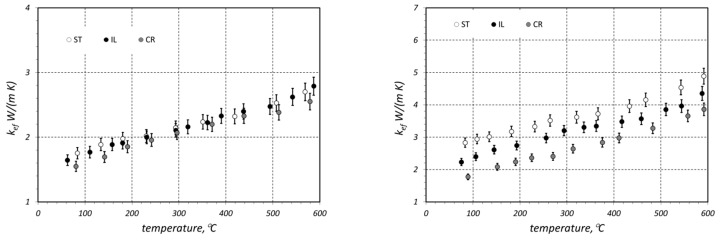

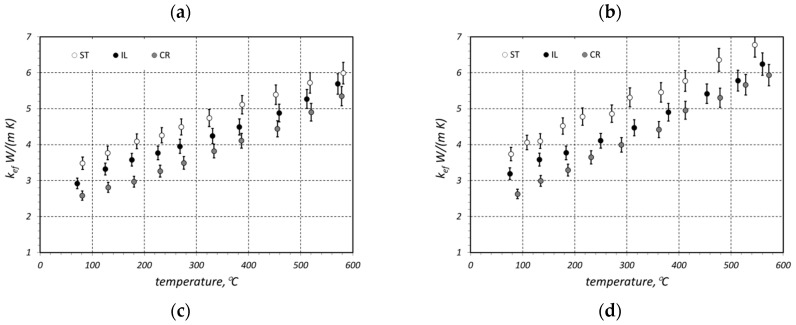
Measured values of the *k_ef_* coefficient obtained for samples made of (**a**) 10 mm bars, (**b**) 20 mm bars, (**c**) 30 mm bars, and (**d**) 40 mm bars.

The results of the *k_ef_* measurements are presented in Figure 4. The maximum measurement uncertainty (*δk_ef_*) was estimated from an error propagation equation [37], and its maximal value was 4.7% [35]. The parameter *δk_ef_* is marked in the graphs by error bars. Each of the graphs presents the results obtained for the three samples (with different arrangement) made of bars with the same diameter. Therefore, comparison of the results presented in one diagram reveals the effect of sample arrangement on the value of *k_ef_*. Except for the samples made of bars with diameter of 10 mm, the results obtained for other cases are unequivocal. The greatest value of *k_ef_* coefficient was observed for staggered (ST) samples, whereas the lowest value was found for the crossed (CR) arrangement. In the latter type, the bars from individual layers are in contact along the whole length, with one bar in contact with two bars from another layer. In the CR samples, due to the crossing of the bars, the contact occurs on many-times smaller, discrete surfaces. The intermediate values of the effective conductivity were obtained for the in-line samples (IL), where bars were in contact along the whole length. However, in this case, each bar from one layer is in contact with only one bar from the next layer. These results demonstrate that contact conduction, with its intensity in granular materials depending on the contact surface area between the adjacent particles, plays a substantial role in the complex heat flow that occurs in bar bundles.

The lack of clear differentiation between individual arrangements in the case of the samples made of 10 mm bars results from the fact that these bars were substantially less rectilinear. This defect caused the bars in staggered and in-line beds to be in contact not along the whole length but only in insignificant sections. Consequently, the arrangement of these bars did not have a significant effect on thermal contact conduction and on the value of *k_ef_* coefficient.

The values of the *k_ef_* coefficient are in the range of 1.5–6.9 W/(m·K). The observed increase in *k_ef_* with temperature for all the samples has a linear character. For this reason, the temperature changes in this parameter for each case were approximated with the linear relationships:

(2) $$k_{ef}\left(t\right)=A_{0}+A_{1}\;t,$$

The values of *A*_0_ and *A*_1_ coefficients from Equation (2) obtained for individual samples are presented in Table 1. The goodness-of-fit equation to the measurement results is expressed with the coefficient of determination *R*^2^, which is also presented in Table 1. The values of *R*^2^ close to one confirm a good match.

## 3. Analytical Model

**Figure 5:**
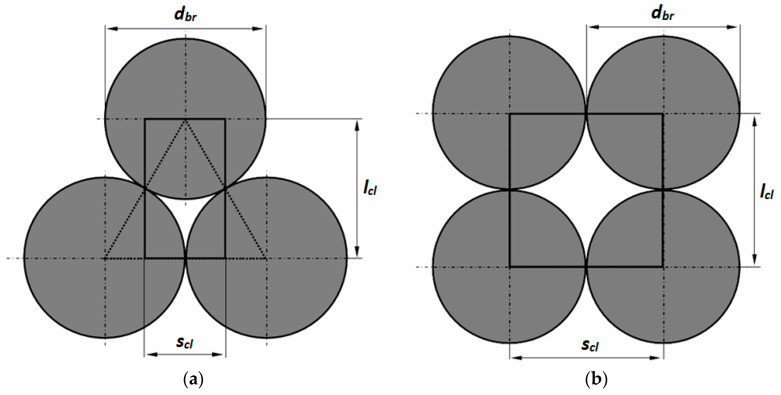
Unit cells defined for the analyzed medium with (**a**) staggered arrangement and (**b**) in-line arrangement.

The equations determined for individual samples described by dependence (2) make it possible to calculate thermal contact conductance *h_ct_*. For this purpose, the special mathematical model of complex heat flow in the bar bed was used. It is assumed that in the analyzed system, heat is transferred by conduction in the section of individual bars, conduction in gas, thermal radiation between the surfaces of bars, and contact conduction. Due to small dimensions of the voids, free convection can be omitted [38]. The starting point of this analysis was the geometrical model of the considered medium in the form of a unit cell. Such an approach is commonly used in the theory of porous and granular materials [21,22,39]. The unit cells defined for the staggered and in-line arrangements are presented in Figure 5. It should be noted that the cell in Figure 5b is also used for crossed structure analysis. This is possible because the crossed structure is very similar to the in-line arrangement. The only difference lies in the fact that in the in-line structure, bars from all layers are arranged parallel to each other, while for the crossed structure, bars from consecutive layers are perpendicular to each other. From the perspective of heat transfer, this only affects the contact conduction because it changes the contact area between the adjacent bars. Therefore, for the heat transfer analysis in the crossed structure, the cell presented in Figure 5b can be utilized. 

The analysis of heat transfer mechanisms which take place in the defined unit cell is based on the thermal resistance concept. It is assumed that in these cells, one-dimensional heat transfer occurs in the vertical direction (along the cell dimension which has been denoted as (*l_cl_*) in Figure 5). Parameter (*s_cl_*) in Figure 5 denotes the width of the unit cell. This quantity determines the heat transfer surface area in the unit cell.

The total thermal resistance of the medium *R_to_* is assumed as a series connection of the conduction resistance in the bars *R_br_* and void thermal resistance *R_vo_*:

(3) $$R_{to}=R_{br}+R_{vo}.$$

The resistance *R_vo_* is calculated as a parallel connection of the thermal contact resistance *R_ct_*, conduction resistance in gas *R_gs_*, and radiation resistance *R_rd_*:

(4) $$R_{vo}=\left(R_{ct}^{-1}+R_{gs}^{-1}+R_{rd}^{-1}\right){\;}^{-1},$$

The value of *R_to_* resistance can be calculated using the definition of conduction resistance of the plane wall [40]:

(5) $$R_{to}=\frac{l_{cl}}{k_{ef}},$$

where *l_cl_* is a cell dimension in the direction of heat flow.

Using Equations (3)–(5), it is possible to calculate the *R_to_* resistance, which is the inverse of thermal contact conductance *h_ct_*. After several rearrangements, the following dependence was obtained:

(6) $$h_{ct}=\left(\frac{1}{\frac{l_{cl}}{k_{ef}}-R_{br}}-\frac{1}{R_{gs}}-\frac{1}{R_{rd}}\right).$$

The method of calculating the resistances *R_br_* and *R_gs_* has been described in the work [41]. These resistances take into consideration the thermal conductivity of bar material *k_br_* and gas *k_gs_*, which fills the voids. The formulated model assumes that the values of the enumerated coefficients change in the temperature function, which is shown by the following equations:

(7) $$k_{br}=1.24\cdot 10^{-8}t^{3}-3.26\cdot 10^{-5}t^{2}-1.19\cdot 10^{-2}t+51.35,$$

(8) $$k_{gs}=-2.88\cdot 10^{-8}t^{2}+8.05\cdot 10^{-5}t+0.024.$$

Equation (7) concerns the thermal conductivity of low-carbon steel with 0.2% carbon content. Bars used during the measurements of the effective thermal conductivity were made of this grade of steel (S235JRH). Equation (8) describes the changes in the thermal conductivity of air. Both equations were determined through approximation of the tabular data available in the literature [42,43].

Radiation resistance *R_rd_* from Equation (6) is described by the following formula [44]:

(9) $$R_{rd}=\frac{X_{cl}}{4\;\sigma_{c}\;T_{m}^{3}},$$

where *σ_c_* is the Stefan–Boltzmann constant, *T_m_* is mean absolute temperature of the considered medium, and *X_cl_* is radiation exchange coefficient. This parameter was calculated with the use of the following relations [44]:

(10) $$X_{cl}=0.955\frac{1.5-0.5\varepsilon }{\varepsilon },$$

(11) $$X_{cl}=0.637\frac{2-0.43\varepsilon }{\varepsilon }.$$

Equation (10) refers to the medium (bar bed) with staggered arrangement, while Equation (11) refers to the beds with in-line and crossed arrangements. When calculating the *X_cl_* coefficient, it is necessary to take into account temperature changes in bar emissivity (*ε*), which is described by the dependence determined by means of experimental investigations, (Equation (12)) [44]:

(12) $$\varepsilon (t)=0.0002\;\left(T_{m}-273\right)+0.64.$$

## 4. Results and Discussion

**Figure 6:**
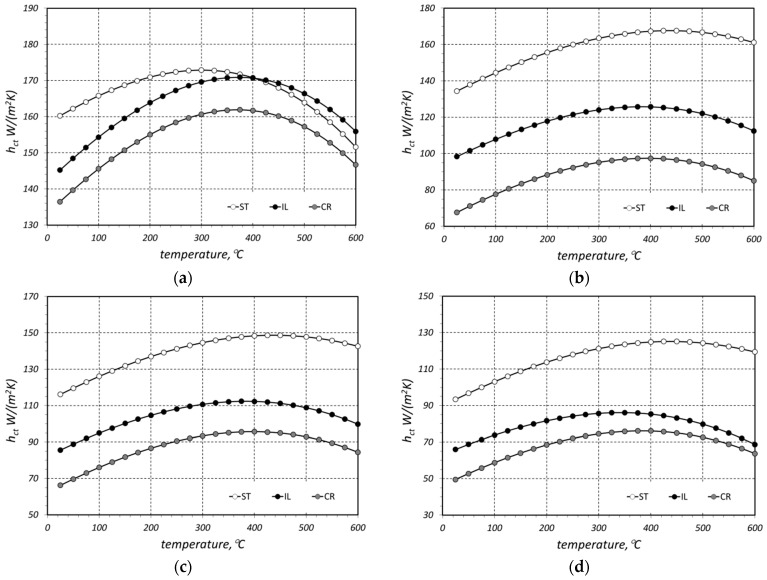
Modelled values of the thermal contact conductance *h_ct_* of the bar beds made of (**a**) 10 mm bars, (**b**) 20 mm bars, (**c**) 30 mm bars, and (**d**) 40 mm bars.

The modelled values of the thermal contact conductance *h_ct_* of the analyzed bar beds are presented in Figure 6. The whole value range of this parameter is relatively broad, from 50 to 173 W/(m^2^·K). It can be observed for all the considered cases that the maximum value of the *h_ct_* coefficient is obtained at a temperature of around 400 °C. At this stage of the investigations, it is not possible to explain this tendency unequivocally due to the complexity of the contact conduction phenomenon. This results from the fact that the value of the *h_ct_* parameter is a function of many different parameters, for example, surface microhardness, Young’s modulus, Poisson ratio, surface roughness, thermal conductivity of bars which are in contact, or mechanical pressure [45,46,47,48]. The values of all the enumerated factors change with a temperature increase, which in consequence causes a certain change in the *h_ct_* value. This problem will be subject of further investigations by the authors.

For further analysis, the obtained results were approximated with regression functions in the form of a second-degree polynomial:

(13) $$h_{ct}\left(t\right)=B_{0}+B_{1}\;t+B_{2}\;t^{2},$$

The values of the *B*_0_, *B*_1,_ and *B*_2_ coefficients from Equation (13) obtained for individual samples are presented in Table 2. The table also contains values of the *R*^2^ coefficient, which confirm a good fit of the function to the approximated results.

Based on the determined regression equations, for each sample with the analyzed temperature range, the minimum, mean, and maximum values of the *h_ct_* coefficient were calculated. These results are shown in Table 3. The last column presents the difference between the maximum and minimum values, which is denoted as Δ*h_ct_*.

Using the *h_ct-mean_* values obtained for individual samples, the influence of the bar diameter on the intensity of contact conductance was analyzed. As can be seen in Table 3, the samples of 40 mm bars have the lowest values of *h_ct-mean_*. Using these values, we introduced three parameters, which are defined with the following equations:

(14) $$h_{ct-10/40}=\frac{h_{ct-10}}{h_{ct-40}},$$

(15) $$h_{ct-20/40}=\frac{h_{ct-20}}{h_{ct-40}},$$

(16) $$h_{ct-30/40}=\frac{h_{ct-30}}{h_{ct-40}}.$$

The values of these parameters are collated in Table 4. In the case of the samples made of 10 mm bars, the crossed samples have the highest value and the staggered ones have the lowest value. In case of the 20 and 30 mm bars, the in-line samples have the highest values. Data from Table 4 show that, apart from the bar arrangement in the sample, the bar diameter also has a big influence on the contact conductance.

A similar procedure was used in relation to the sample arrangement. The crossed samples are characterized by the lowest *h_ct_* values. Using these values, the following parameters were defined:

(17) $$h_{ct-ST/CR}=\frac{h_{ct-ST}}{h_{ct-CR}},$$

(18) $$h_{ct-IL/CR}=\frac{h_{ct-IL}}{h_{ct-CR}}.$$

The values of these parameters are presented in Table 5. As can be seen, the change in bar arrangement from the crossed one to the staggered one—apart from the 10 mm bars—increases contact conductance from 60% (30 mm bars) to 78% (20 mm bars). For the 10 mm bars, we can observe a 9% increase. In the case of changing the crossed arrangement to the in-line one, the increase in the *h_ct_* coefficient value ranges from 16% (40 mm bars) to 32% (20 mm bars). Similarly, as in the previous situation, for the 10 mm bars, this increase was only 6%.

Using the *B*_0_, *B*_1_, and *B*_2_ coefficients from Table 2, a generalization of the obtained results in relation to bar diameter was performed. We analyzed how the above-mentioned coefficients change in the bar diameter function for individual samples. It was established that changes in the *B*_0_ and *B*_2_ parameters can be described with linear equations in the following form (where diameter *d_br_* is expressed in mm):

(19) $$B_{0-ST}\left(d_{br}\right)=-2.21\;d_{br}+176.7,$$

(20) $$B_{0-IL}\left(d_{br}\right)=-2.48d_{br}+155.8,$$

(21) $$B_{0-CR}\left(d_{br}\right)=-2.61\;d_{br}+140.0,$$

(22) $$B_{2-ST}\left(d_{br}\right)=\left(0.0057\;d_{br}-2.1\right)\cdot 10^{-4},$$

(23) $$B_{2-IL}\left(d_{br}\right)=\left(0.0042\;d_{br}-2.4\right)\cdot 10^{-4},$$

(24) $$B_{2-CR}\left(d_{br}\right)=\left(0.0052\;d_{br}-2.4\right)\cdot 10^{-4},$$

The *B*_1_ coefficients in the bar diameter function change insignificantly; therefore, for each bar arrangement, a constant, averaged value of *B*_1_ was adopted. Using the data from Table 2, the following values were obtained:

(25) $$B_{1-ST}=\frac{0.117+0.174+0.169+0.165}{4}=0.156,$$

(26) $$B_{1-IL}=\frac{0.169+0.173+0.167+0.166}{4}=0.168,$$

(27) $$B_{1-CR}=\frac{0.169+0.181+0.176+0.169}{4}=0.173.$$

By substituting the Expressions (19)–(27) into Equation (13), we obtained generalized approximation equations describing changes in the thermal contact conductance simultaneously in the temperature and bar diameter function:

(28) $$h_{ct-ST}\left(t\right)=-2.21\;d_{br}+176.7+0.156\;t+\left(0.0057\;d_{br}-2.1\right)\cdot 10^{-4}\;t^{2},$$

(29) $$h_{ct-IL}\left(t\right)=-2.48\;d_{br}+155.8+0.168\;t+\left(0.0042\;d_{br}-2.4\right)\cdot 10^{-4}\;t^{2},$$

(30) $$h_{ct-CR}\left(t\right)=-2.61\;d_{br}+140.0+0.173\;t+\left(0.0052\;d_{br}-2.4\right)\cdot 10^{-4}\;t^{2}.$$

This may raise the question of why different equations, Equations (6) and (28)–(30), were used to calculate the *h_ct_* parameter. Formula (6) allowed for the calculation of the *h_ct_* coefficient based on the results of the experimental studies (measurements of the *k_ef_* coefficient). In this case, the values of *k_ef_* were approximated using Equation (2) for the *h_ct_* parameter calculations. This approach resulted in the outcomes presented in Figure 6. Subsequently, these results underwent a further analysis with the aim of deriving generalized relationships in the form of Equations (28)–(30). These equations, for each of the three considered bar arrangements, enabled the calculation of the *h_ct_* coefficient as a function of temperature and bar diameter. Equation (6) did not provide this capability; it was only used as an auxiliary tool to derive Equations (28)–(30).

**Figure 7:**
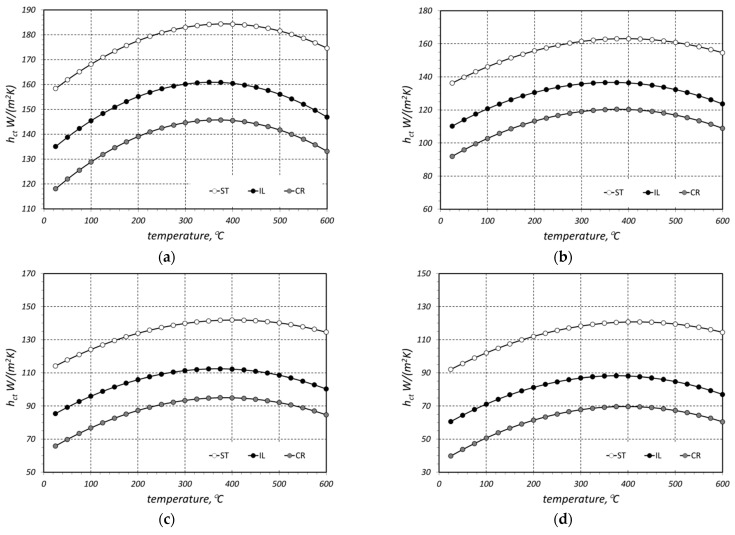
Values of the thermal contact conductance *h_ct_* calculated with the use of Equations (27)–(29) for (**a**) 10 mm bars, (**b**) 20 mm bars, (**c**) 30 mm bars, and (**d**) 40 mm bars.

The results of the *h_ct_* calculations obtained with the use of Equations (28)–(30) are shown in Figure 7. As can be seen, these values differ from the results in Figure 6. In order to define the exact discrepancy between the results from both figures, we used the *δh_ct_* parameter, which was defined with the following dependence:

(31) $$\delta h_{ct}=\frac{\left|h_{ct-m}-h_{ct-ap}\right|}{h_{ct-m}}\times 100\%,$$

where *h_ct-m_* modelled values of the thermal contact conductance and *h_ct-ap_* modelled the thermal contact conductance obtained on the basis of approximation Equations (28)–(30). The maximum values of the *δh_ct_* parameter and the ones which were averaged in relation to temperature are presented in Table 6.

This juxtaposition shows that the obtained discrepancies of the results for individual cases are diverse. The highest values of *h_ct-max_* and *δh_ct-mean_* equal to 36.1% and 27.3%, respectively, were obtained for the 20 mm bars with a crossed arrangement. For other calculation cases, the values of both parameters did not exceed 20%. When analyzing data from Table 6, it must be stated that from a practical point of view, the most significant results are the ones for the staggered samples. As can be seen in Figure 1, that is the structure of the treated bar bundles. The in-line and crossed arrangements are in this case purely theoretical. The *δh_ct-mean_* values for the staggered case for further bar diameters equal 6.5%, 1.9%, 3.5%, and 2.5%. This indicates that the values of the *h_ct_* coefficient obtained with the use of the approximation Equation (28) are relatively compatible with the results of the precise calculations (obtained with the use of a model based on the analysis of thermal resistances).

**Figure 8:**
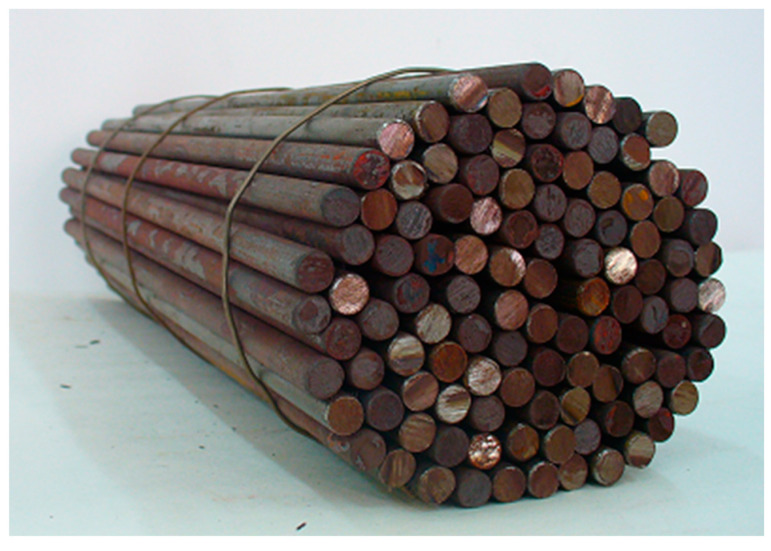
The bar bundle model used in laboratory research exhibits an irregular arrangement, as seen in this case, which is a combination of staggered and in-line arrangements.

To summarize the conducted research, it was decided to clarify two issues relating directly to industrial practice. The first is the potential industrial applications of various bar arrangements. To address this issue, a photograph of a 10 mm bar bundle was utilized, as shown in Figure 8. This is a model bundle used for laboratory studies. It should be emphasized that bundles of bars heated under industrial conditions, similar to the bundle depicted in Figure 8, often exhibit an irregular arrangement due to the absence of a maximum bar-packing standard. The geometry of such bundles combines staggered and in-line arrangements. In cases where the bars within the bundle are laid out haphazardly, they may even intersect each other. Therefore, depending on the regularity of the bar placement in the bundle, and, consequently, its packing density, all three of the considered arrangements can find industrial applications. For meticulously arranged bundles, the staggered arrangement is the most suitable, while for loosely packed bundles, the crossed arrangement might be the most appropriate. Thus, from an industrial application perspective, each of the analyzed arrangements can be considered. However, to enhance heat transfer intensity, the best solution is the staggered arrangement, and this layout should be pursued when preparing the charge for heating in the furnace.

The second issue is which thermal treatments are to be applied according to the temperatures selected in the study. The most common heat treatment for steel bars is recrystallization annealing, typically applied after prior plastic processing. The objective of this operation is to improve the plastic properties of the steel. It is carried out below the austenite transformation temperature (723 °C) but above the recrystallization temperature. For steel, depending on its chemical composition, this temperature range is from 400 °C to 700 °C. Therefore, the process of heating the bundle of bars proceeds from room temperature to around 700 °C. Below approximately 500 °C, the key heat exchange process, in terms of intensity, is conductive heat transfer. However, as previously demonstrated by the authors in their earlier publications [11,38,41,44], above 500 °C, the most significant influence on the heating process of the bundle is thermal radiation. Thus, the analysis of conductive heat transfer within the temperature range chosen by the authors, i.e., from 20 to 600 °C, is highly justified. As seen in Figure 6, after surpassing the temperature of 500 °C, the intensity of thermal contact conduction begins to decrease. When considering the temperature at which the heat treatment of bars is carried out, one must take into account that the heat transfer process in a bundle of bars is a complex phenomenon. It comprises a combination of conduction (within the bars and the gas), contact conduction, and thermal radiation. The changes in the intensity of each of these heat transfer phenomena with the change in the charge temperature occur completely differently. This is a complex issue related to the qualitative heat exchange in a bundle and will be the subject of further analysis.

## 5. Conclusions

Using the results of the experimental investigations and the model of complex heat transfer based on the analysis of thermal resistances, we determined the values of the thermal contact conductance *h_ct_* for a bed of steel bars. It was determined that depending on factors such as temperature, diameter, and arrangement of the bars, the values of the *h_ct_* parameter are within the range from 50 to 173 W/(m^2^·K). Next, thanks to the approximation of the results of the model calculations for each bar arrangement, we derived generalized approximation equations which enabled the calculation of the value of the *h_ct_* coefficient for the packed bar bundles simultaneously in the temperature and bar diameter function. The values of the *h_ct_* coefficient obtained with the use of this equation for a bar bed with a staggered arrangement (which is how bundles are packed during heat treatment) depending on the bar diameter, differ from the results of the model calculations by 1.9 to 6.5%. Taking into account the complexity of the analysis issue, the level of discrepancy of the results can be regarded as satisfactory. In connection with this, the derived equation can be used to develop a universal model of the effective thermal conductivity of round bar bundles with arbitrary porosity. Developing such a model will be the next stage of work by the authors in the area of heating bundles of steel bars. Upon its validation, such a model will be a valuable computational tool for the optimization of heat treatment processes.

## Figures and Tables

**Table 1:** The values of the *A*_0_, *A*_1_, and *R*^2^ coefficients from Equation (2) obtained for individual samples.

Bar Diameter, mm	Sample Arrangement	*A*_0_	*A*_1_	*R* ^2^
10	staggered	1.615	0.0018	0.980
	in-line	1.532	0.0020	0.991
	crossed	1.448	0.0020	0.984
20	staggered	2.483	0.0037	0.981
	in-line	2.097	0.0037	0.983
	crossed	1.427	0.0039	0.991
30	staggered	3.117	0.0050	0.997
	in-line	2.586	0.0052	0.983
	crossed	2.032	0.0055	0.993
40	staggered	3.312	0.0063	0.991
	in-line	2.667	0.0061	0.992
	crossed	2.031	0.0069	0.998

**Table 2:** The values of the *B*_0_, *B*_1_, *B*_2_, and *R*^2^ coefficients from Equation (13) for individual samples.

Bar Diameter, mm	Sample Arrangement	*B*_0_	*B*_1_	*B*_2_	*R* ^2^
10	staggered	156.4	0.117	−2.05 × 10^−4^	0.92
	in-line	140.0	0.169	−2.35 × 10^−4^	0.994
	crossed	131.3	0.169	−2.36 × 10^−4^	0.995
20	staggered	129.2	0.174	−1.99 × 10^−4^	0.998
	in-line	93.1	0.173	−2.31 × 10^−4^	0.996
	crossed	62.1	0.181	−2.35 × 10^−4^	0.996
30	staggered	111.3	0.169	−1.93 × 10^−4^	0.998
	in-line	80.6	0.167	−2.25 × 10^−4^	0.997
	crossed	61.0	0.176	−2.25 × 10^−4^	0.997
40	staggered	88.6	0.165	−1.88 × 10^−4^	0.999
	in-line	61.4	0.166	−2.23 × 10^−4^	0.995
	crossed	44.6	0.169	−2.22 × 10^−4^	0.995

**Table 3:** The values of *h_ct-min_*, *h_ct-mean_*, *h_ct-max_*, and Δ*h_ct_* obtained for individual samples.

Bar Diameter, mm	Sample Arrangement	*h_ct-min_*	*h_ct-mean_*	*h_ct-max_*	Δ*h_ct_*
10	staggered	151.6	166.7	172.9	21.3
	in-line	145.2	162.9	170.8	25.6
	crossed	136.5	153.4	161.9	25.4
20	staggered	134.3	158.1	167.6	33.3
	in-line	98.3	117.5	125.7	27.4
	crossed	67.6	88.7	97.4	29.8
30	staggered	116.2	139.5	148.7	32.5
	in-line	85.6	104.4	112.3	26.7
	crossed	66.2	87.1	95.7	29.5
40	staggered	93.3	116.2	125.1	31.8
	in-line	65.9	79.13	86.1	20.2
	crossed	49.5	68.2	76.1	26.6

**Table 4:** Values of the parameters defined by Equations (14)–(16).

Sample Arrangement	*h_ct-10/40_*	*h_ct-20/40_*	*h_ct-30/40_*
staggered	1.43	1.36	1.20
in-line	2.06	1.48	1.32
crossed	2.25	1.30	1.28

**Table 5:** Values of the parameters defined by Equations (17) and (18).

Bar Diameter, mm	*h_ct-ST/CR_*	*h_ct-IL/CR_*
10	1.09	1.06
20	1.78	1.32
30	1.60	1.20
40	1.70	1.16

**Table 6:** The maximum and averaged values of the *δh_ct_* parameter.

Sample Arrangement	*d_br_* = 10 mm		*d_br_* = 20 mm		*d_br_* = 30 mm		*d_br_* = 40 mm	
	*δh_ct-max_,*	*δh_ct-mean_*	*δh_ct-max_,*	*δh_ct-mean_*	*δh_ct-max_,*	*δh_ct-mean_*	*δh_ct-max_,*	*δh_ct-mean_*
	*%*							
staggered	15.2	6.5	4.3	1.9	5.6	3.5	4.1	2.5
in-line	7.0	5.9	12.2	9.9	1.3	0.6	12.1	4.2
crossed	13.5	10.4	36.1	27.3	1.0	0.6	19.6	9.9

## Data Availability

The data presented in this study are available in: Wyczółkowski, R. Experimental Investigations of Effective Thermal Conductivity of the Selected Examples of Steel Porous Charge. Solids 2021, 2, 420–436. https://doi.org/10.3390/solids2040027.
